# Esophagitis Dissecans Superficialis in a 47-Year-Old Female With Dysphagia and Epigastric Pain: A Case Report

**DOI:** 10.7759/cureus.97274

**Published:** 2025-11-19

**Authors:** Rachel G Droznik, Michelle Y Zhu, Eleanor C Fung

**Affiliations:** 1 Department of Surgery, University at Buffalo Jacobs School of Medicine and Biomedical Sciences, Buffalo, USA; 2 Department of Pathology, University at Buffalo Jacobs School of Medicine and Biomedical Sciences, Buffalo, USA

**Keywords:** benign esophageal disease, dysphagia, esophagitis dissecans, esophagitis dissecans superficialis, esophagogastroduodenoscopy (egd), esophagus, sloughing esophagitis

## Abstract

Esophagitis dissecans superficialis (EDS) is an infrequently diagnosed benign esophageal disease that presents with sloughing of the esophageal mucosa into the esophageal lumen. It often presents after esophageal irritation but may also occur idiopathically. Treatment is approached by cessation of the offending agent and initiation of a proton pump inhibitor (PPI). We present the case of a 47-year-old female with persistent epigastric pain and dysphagia despite twice-daily PPI use. The patient had several known risk factors for EDS. Diagnosis was subsequently confirmed by upper endoscopy and histopathological analysis. The patient was advised to continue PPI treatment indefinitely.

## Introduction

Esophagitis dissecans superficialis (EDS) is a rare and underdiagnosed benign endoscopic finding characterized by the sloughing of the esophageal mucosa into the esophageal lumen, most commonly in the middle and distal portions of the esophagus. The etiology of EDS is likely multifactorial and can be attributed to smoking, alcohol, medications, bullous skin conditions, or idiopathic causes. One study estimated the incidence to be as low as 0.03% [1]. Symptoms include epigastric pain and dysphagia, making it difficult to distinguish from gastroesophageal reflux disease or eosinophilic esophagitis on clinical presentation. It may be detected on a routine esophagogastroduodenoscopy (EGD) performed for unrelated symptoms or after patients present in emergent settings. Biopsy shows sloughing of the esophageal mucosa, distinguishing it from other more common esophageal pathologies [2]. Due to its rarity, varied presentation, and unclear pathogenesis, EDS remains poorly understood. We present a case of EDS diagnosed on EGD following dysphagia and epigastric pain refractory to proton pump inhibitors (PPIs).

## Case presentation

A 47-year-old female with a history of prior alcohol use disorder, hypertension, open gastric bypass 20 years prior, and bipolar disorder presented for elective EGD to evaluate symptoms of dysphagia. At the time of the diagnostic endoscopy, she reported several months of dysphagia and daily postprandial epigastric pain refractory to twice-daily pantoprazole. She had not had any prior EGD evaluations.

The patient was a non-smoker and disclosed ongoing abstinence from alcohol, supported by use of disulfiram therapy. Medication use included 81 mg aspirin daily, valsartan, propranolol, gabapentin, fluoxetine, lamotrigine, brexpiprazole, trazodone, and bupropion. Aspirin and disulfiram were discontinued seven days before the procedure. The patient denied any other chronic non-steroidal anti-inflammatory drug (NSAID) use. Laboratory assessments leading up to the EGD were unremarkable (Table 1).

**Table 1:** Laboratory findings.

Parameter	Result	Reference range
White blood cell count	5.5 × 10^9^/L	4.0–10.5 × 10^9^/L
Red blood cell count	4.13 × 10^12^/L	4.20–5.40 × 10^12^/L
Hemoglobin	11.1 g/dL	12.0–16.0 g/dL
Hematocrit	35.7%	37.0–47.0%
Mean corpuscular volume	86.4 fL	78.0–100.0 fL
Mean corpuscular hemoglobin	26.9 pg	28.0–34.0 pg
Mean corpuscular hemoglobin concentration	31.1 g/dL	32.0–36.0 g/dL
Red cell distribution width coefficient of variation	19.30%	11.5–14.0%
Platelet count	307 × 10^9^/L	150–459 × 10^9^/L
Mean platelet volume	9.2 fL	9.0–12.0 fL
Basophils (auto)	1.30%	≤3.0%
Eosinophils (auto)	5.90%	≤6.0%
Neutrophils (auto)	47.8%	38.0–77.0%
Lymphocytes (auto)	30.5%	20.0–48.0%
Monocytes (auto)	14.3%	≤12.0%
Sodium	140 mmol/L	135–145 mmol/L
Potassium	4.1 mmol/L	3.5–5.3 mmol/L
Chloride	107 mmol/L	96–110 mmol/L
Carbon dioxide	24 mmol/L	20–32 mmol/L
Anion gap	9 mmol/L	5–15 mmol/L
Blood urea nitrogen	13 mg/dL	5–25 mg/dL
Creatinine	0.96 mg/dL	0.4–1.40 mg/dL
Estimated glomerular filtration rate	73 mL/min/1.73m²	>59 mL/min/1.73m²
Glucose	87 mg/dL	60–100 mg/dL
Calcium	8.5 mg/dL	8.5–10.5 mg/dL
Total bilirubin	0.3 mg/dL	0.2–1.2 mg/dL
Albumin	3.9 g/dL	3.5–5.0 g/dL
Total protein	6.2 g/dL	6.0–8.0 g/dL
Alkaline phosphatase	66 U/L	30–140 U/L
Aspartate aminotransferase	15 U/L	5–50 U/L
Alanine aminotransferase	19 U/L	5–50 U/L
International normalized ratio	0.88	-
Prothrombin time	12.2 seconds	11.0–15.0 seconds
Partial thromboplastin time	29.3 seconds	25.0–34.0 seconds

**Figure 1:**
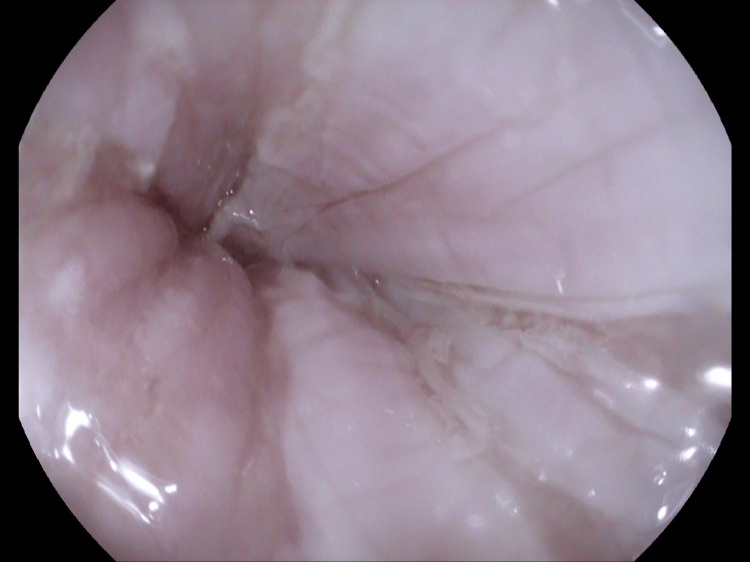
Endoscopy at the distal esophagus (near gastroesophageal junction) shows sloughing tissue.

**Figure 2:**
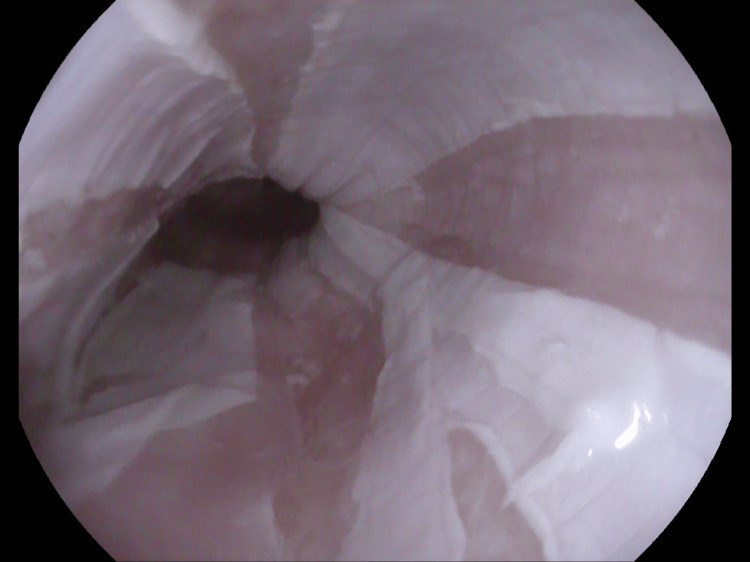
Sloughing continues in mid-esophagus.

**Figure 3:**
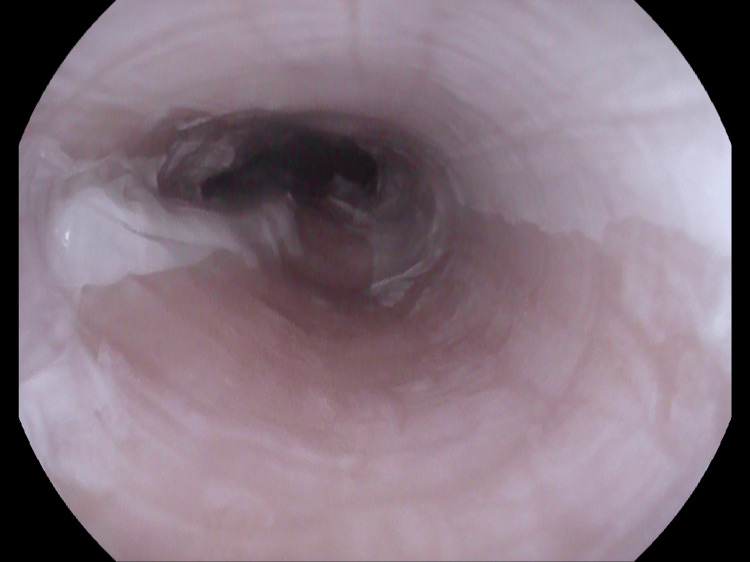
Proximal esophagus.

Endoscopy revealed vertical strips of white sloughing mucosa throughout the esophagus, consistent with a diagnosis of EDS (Figures 1-3). Biopsies were obtained in the mid and distal esophagus.

**Figure 4:**
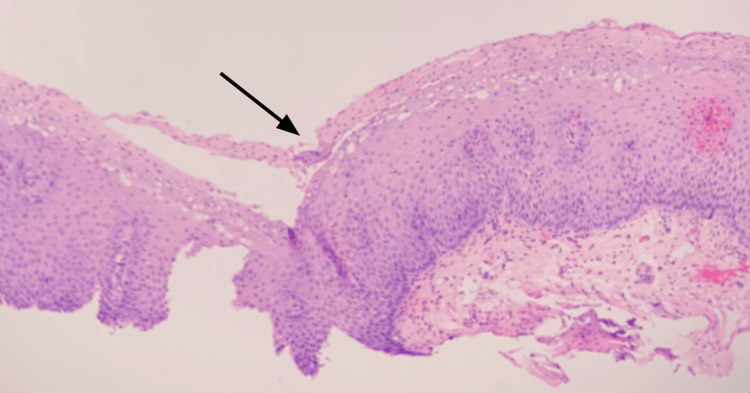
Histopathologic examination of the esophagus showed the superficial layers of the esophageal epithelium are necrotic and have separated from the underlying viable epithelium. Pyknotic nuclei on superficial layers resemble parakeratosis (hematoxylin and eosin, 100×). [3,4].

**Figure 5:**
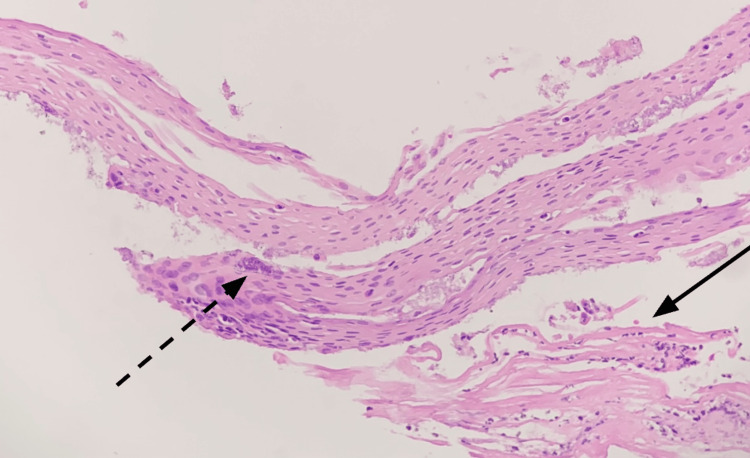
Bacterial colonies are present within detached strips of epithelium (dashed arrow). Inflammatory infiltrate includes a mixture of neutrophils and eosinophils (solid arrow) (hematoxylin and eosin, 200×). [3,4].

Biopsies taken during the procedure were submitted for histopathologic examination and revealed strips of benign squamous epithelium with changes suggestive of gastric reflux. Additional specimens from the distal esophagus near the gastroesophageal junction showed detached strips of squamous epithelium with colonized bacteria admixed with neutrophilic infiltrates and some eosinophils (Figures 4, 5).

The patient tolerated the procedure well without any complications. She was advised to continue her PPI and to follow up with her primary gastrointestinal physician in three weeks. No further follow-up data were available.

## Discussion

The presentation of EDS is variable and ranges from asymptomatic to dysphagia, odynophagia, epigastric pain, and, less frequently, upper gastrointestinal bleeding [1,5]. Diagnosis requires a combination of patient history, endoscopic evaluation, and multiple esophageal biopsies with histopathological confirmation, as it can be easily overlooked depending on its presentation. In some cases, EDS can appear candidal or as non-specific esophagitis and can be refractory to medication when treated as such [6,7]. Biopsies should be performed to rule out malignant manifestations of esophageal disease, including eosinophilic esophagitis and squamous cell carcinoma. While eosinophilic esophagitis may have a similar clinical presentation to EDS, including dysphagia and gastric reflux, microscopic evaluation would reveal numerous eosinophils in the esophageal epithelium [8]. Histological assessment of EDS often shows intraepithelial splitting and parakeratosis. Pathologists must distinguish the true intraepithelial splitting from mechanically induced splitting to aid in the diagnosis [2].

The exact etiology of the condition remains unclear, but it is believed to stem from an insult to the esophageal mucosa, inducing changes that cause sloughing of the mucosa. The insult is often physical, chemical, or thermal, but can also be immunological, as seen in bullous pemphigoid, pemphigus vulgaris, and celiac disease [9-11]. Several medications have an established connection with EDS, including NSAIDs, novel direct oral anticoagulants, bisphosphonates, and selective serotonin reuptake inhibitors (SSRIs) [2,12-15]. Non-medication chemical ingestion and physical irritants such as alcohol and smoking have also been associated with EDS [12,15]. Although irritants and immunological diseases can be implicated, many cases remain idiopathic with no identifiable risk factors.

Our patient possessed multiple factors that put her at risk for EDS, including daily aspirin and fluoxetine use, and a history of chronic alcohol use. The exact etiology of her esophagitis dissecans is difficult to determine due to these multiple risk factors and the undiscovered, likely multifactorial nature of EDS. Although it is not known to us when her alcohol consumption began, it is possible that it was an inciting factor that was then exacerbated by her use of NSAIDs and SSRIs.

Treatment of EDS is dependent on the inciting factor. Cessation of the offending agent is indicated when applicable. Additional treatment is focused on the initiation or continuation of PPIs, with one study showing a complete resolution of EDS in four of five patients on follow-up EGD, and the fifth having mild esophagitis [12]. However, there is some evidence that PPIs may prevent further damage to the mucosa rather than treating the condition, as there have been several cases of patients using PPIs at the time of EDS presentation [16]. This may explain our patient’s persistent symptoms despite PPI use. Follow-up EGD may be performed four weeks after the initial diagnosis to determine further management of PPIs [17]. EGD performed in such patients has shown resolution in as short as eight weeks. In clinical practice, few patients undergo repeat EGD, and discontinuation of the medication may be based on the resolution of symptoms and physician’s discretion [16]. Prognosis is favorable, and complications are extremely rare [2].

## Conclusions

EDS is a rare benign esophageal condition whose presentation often appears alarming on evaluation. It is often idiopathic, but can also be caused by an insult to the esophageal mucosa. Diagnostic workup requires excluding other non-benign esophageal presentations such as eosinophilic esophagitis and squamous cell carcinoma. Our patient had multiple risk factors that increased her risk of developing this disease, including a history of alcohol use disorder and use of NSAIDs and SSRIs at the time of evaluation. Although there are no clear guidelines on the treatment of EDS, initiation or continuation of PPIs is advised in most cases, as was done with our patient.
